# The impact of communication abilities on independence in everyday life—a cross-sectional study of adults with cerebral palsy

**DOI:** 10.3389/fresc.2026.1710509

**Published:** 2026-05-13

**Authors:** Ellen Backman, Kate Himmelmann

**Affiliations:** 1 Department of Social Work, Marie Cederschiöld University, Stockholm, Sweden; 2 Division of Speech and Language Pathology, Institute of Neuroscience and Physiology, University of Gothenburg, Gothenburg, Sweden; 3 Department of Pediatrics, Institute of Clinical Sciences, Sahlgrenska Academy, University of Gothenburg, Gothenburg, Sweden

**Keywords:** AAC, cerebral palsy, complex communication needs, independence, transition to adulthood

## Abstract

**Introduction:**

Communication is imperative for social participation and for being an independent and autonomous adult. Despite this, little is known about how communication abilities in adults with childhood-onset disabilities impacts independence. This study explores nine everyday life domains and the associations of independence with communication effectiveness and speech impairment in adults with cerebral palsy (CP).

**Methods:**

A cross-sectional study was conducted in western Sweden. Transitioning to and establishing independence in adulthood was assessed using the Rotterdam Transition Profile. Associations with communication effectiveness and speech production, as well as the use of augmentative and alternative communication (AAC), was explored. A total of 139 participants born 1979 - 1998 (74 males, 62 females, and 3 non-binary; mean age 30y 1mo, range 18 - 43y) were included of which 28% were described to have complex communication needs.

**Results:**

Over 70% of all participants reported the highest level of independence in the domains “Employment and education” and “Finances”. In other domains, for example “Relationships”, less than 50% reported at this level. All participants reported low levels of independence in the domains “Service and aids” and “Sexuality”. Greater independence in all everyday life domains was associated with having effective communication. Impaired speech was significantly associated with less independence in all domains, except “Finances”. The use of AAC supported independence in the domains “Leisure” and “Relationships”.

**Discussion:**

Communication is a crucial factor to consider in both research and practice to enhance the understanding of independence in persons with cerebral palsy. Complex communication needs are common among adults with CP and must be addressed — regardless of motor function — to support successful transition to adulthood and living the life one wants.

## 1 Introduction

Adulthood involves having responsibility for personal finances, health, relationships, and leisure (1). This responsibility can be demanding for persons with childhood-onset disabilities such as cerebral palsy (CP), partly due to limited societal support, and lack of opportunities for participation in everyday activities, and partly due to specific impairment(s) and the need to acquire additional health literacy and health maintenance skills (2–4). Accomplishing tasks independently, without support from others, may be unrealistic for many people with CP; however, autonomy, where one makes decisions in and about one's own life may be more achievable and relevant. Conceptually, one may grow in autonomy by making decisions for oneself, while still requiring assistance from other people to complete some tasks (5). Assuming adult roles and responsibilities can pose even greater challenges for persons with CP and co-occurring communication disorders and complex communication needs (CCN) (6).

CP, the most common motor disability in childhood, is a group of disorders that affect a person's ability to move, maintain balance, and posture (7). CP may be accompanied by disturbances of sensation, communication, and visual and auditory perception. Commonly co-occurring health conditions also include intellectual disability, autism spectrum disorder, and attention and deficit/hyperactivity disorder (ADHD) (8). Complex communication needs, comprising difficulty in understanding the speech and language of others and/or in using speech and writing to express oneself, relate closely to intellectual function (9–11) and occur in various forms and levels of severity in some persons following CP, ultimately affecting the ability of an individual to communicate within everyday situations. In Swedish adults with CP, nearly 20 per cent have been reported to have a CCN/disorder of speech, impacting their ability to communicate (10). Dysarthria, a motor speech disorder, comprising difficulty in articulating and/ or controlling speech rhythm, has been noted as the most prevalent accompanying impairment in children/young people with CP, with up to 90 per cent displaying dysarthric symptoms (12, 13). Data from 3,000 children included in the Swedish national follow-up register CPUP (cpup.se) reported that 50 per cent of the children alternated independently and effectively between being a sender and receiver of information with most people in most environments, and 14 per cent used various forms of image-based augmentative and alternative communication (AAC), such as communication boards or speech generating devices (14).

Transition from child- to adulthood is a developmental process which, besides taking increased responsibility in everyday life, includes adopting adult roles, building and strengthening one's own identity and reaching independence from parents (1, 15). It typically involves maintaining a home, establishing oneself in the labor market, and becoming involved in the community. Previous research details increased challenges for adolescents and young adults with CP during this life stage, particularly with their sense-of-self, in building relationships, and in engaging fully in key life situations (16–20). For example, young adults with CP live with their parents in the family home to a higher extent than their peers, and are more frequently un-employed (19, 20). They are often dependent on parental support, both financially and with activities of daily living (20), and experience difficulties with making new, meaningful friendships (17). Transition processes for young people with CP are often elongated and delayed (18–21), and the degree of physical impairment significantly moderates the level of participation achieved by young people with CP (18, 19). Over the lifespan, there continues to be an interplay between individual and societal factor shaping the life of adults with CP. For example, the presence of epilepsy, walking ability and whether a person is classified as having intellectual disability change over time, underscoring that CP is not a static condition (22). Furthermore, successful outcomes across many life domains for adults with CP are formed by available social and practical support, societal attitudes and expectations toward people with CP, and the accessibility of activities and environments (23, 24).

Communication, the conscious and unconscious social process that refers to the interchange of verbal, non-verbal, or graphic messages from one person to another (25), is of particular importance for social participation and autonomy (20). Communication enables the development and deepening of relationships, giving and receiving information, and being an active citizen (26, 27), and thus plays a key role in the process of transitioning to adulthood (28). Communicative competence (26, 29) is essential for effectively interacting with both familiar and unfamiliar people in a variety of everyday life contexts. These situations are characterized by legal rights and obligations, increased autonomy and responsibility and accountability for actions in adulthood, in contrast to what could be expected during childhood.

Studies exploring transition to and independence in adulthood for persons with CP consistently report information on motor function, and in some cases intellectual disability, presenting CP as predominantly a motor disorder (30). However, studies seldom report aspects of a person's communicative competence or CCN (16, 30) despite the high prevalence of CCN in persons with CP and research showing that childhood-onset communication disorders have enduring negative effects on the development and maintenance of social relationships across the lifespan (31). For example, in a recent scoping review on transition to adulthood for persons with CP, only half of the reviewed studies mentioned the participants' communication abilities, and only 4 participants (0.3%) were described to use some form of AAC (30). Overlooking communication abilities and communicative competence leaves the relative impact of CCN on independence in adulthood poorly understood, as compared to other impairments associated with CP. This limits the capacity of e.g., family members and professionals to provide adequate support for people with CP across everyday life areas, for example in healthcare, occupation, and community engagement.

Thus, the aim of this study was to describe independence within a set of everyday life domains in adults with cerebral palsy (CP) and explore the associations of independence with communication effectiveness and speech impairment. A secondary aim was to describe the use of augmentative and alternative communication (AAC). The following research questions were posed: (1) Does the level of independence across everyday life areas differ between adults with CP and effective communication (considering speech, gestures, facial expressions, and AAC) compared to those with non-effective communication, and if so how?; (2) Does the level of independence across everyday life areas differ between adults with cerebral palsy with unaffected speech compared to those with reduced speech intelligibility, and if so how?; (3) Is the use of AAC associated with independence and if so, how? It was hypothesized that participants with effective communication and unaffected speech would be more independent within the explored everyday life areas, and at a younger age, and that AAC would support independence.

## 2 Materials and methods

### 2.1 Design

This cross-sectional study recruited participants from the CP register of western Sweden. The register includes all persons with CP born since 1954 and who, at the age of 4–8 years, lived in the Swedish counties of Västra Götaland, Jönköping and Halland. The study is part of the project “Living as an adult with cerebral palsy in western Sweden” (32), and was approved by the Regional Ethics review board in Gothenburg 2014-01-16, no. 777-13.

### 2.2 Participants

Data were collected between 2015 and 2023, and potential participants comprised adults from the register aged between 18 and 43 years, meaning they were born between 1979 and 1998. All eligible persons were invited by mail to participate. The invitation was followed by a telephone call and a second letter. Persons who had moved into the area, and therefore not in the register from birth, were invited through habilitation units and patient organizations. The age span, including both the periods typically referred to as “emerging adulthood” (1, 15), and “established adulthood” (33) was chosen to capture any delayed, elongated or absent phases of transition, thereby expanding previous research from a narrow “transition” focus to a broader lifespan perspective on independence for persons with CP. No exclusion criteria were applied. Participants or their legal guardian gave verbal and written consent to take part in the study.

### 2.3 Measures

The focus on communication was applied through adapting the data collection to be inclusive for persons with CCN and through including variables depicting intrapersonal aspects of communicative competence. Demographic and clinical characteristics were used together with the Rotterdam Transition Profile (RTP) questionnaire (25). Information was gathered about gender, age, gross motor function, manual ability, communication, school form, occupation, and CP subtype (classified as unilateral spastic, bilateral spastic, dyskinetic, or ataxic (34). Gross motor function was classified according to the Gross Motor Function Classification System (GMFCS) (35), manual ability was classified according to the Manual Ability Classification System (MACS) (36) and the Communication Function Classification System (CFCS) (37) was used to describe communication. The CFCS provides a description of the effectiveness, clarity, and consistency of everyday communication with familiar and unfamiliar partners. Each level describes characteristic patterns of message transmission and conversational success, enabling standardized comparison of communication abilities in clinical and research settings, Table 1. The classification considers all forms of communication, including speech, gestures, facial expressions, and AAC. The GMFCS, MACS, and CFCS (with a five-level scoring system reaching from Level I, *most able*, to Level V, *least able*) delineate functional ability by focusing on activity and participation as described in the International Classification of Functioning, Disability and Health, ICF (38).

**Table 1:** The communication function classification system (CFCS) (37).

CFCS level	Description
Level I	The person independently and effectively alternates between being a sender and receiver of information^a^ with most people in most environments.
Level II	The person independently alternates between being a sender and receiver with most people in most environments, but the conversation may be slower.
Level III	The person usually communicates effectively with familiar communication partners, but not with unfamiliar partners, in most environments.
Level IV	The person is not always consistent at communicating with familiar communication partners.
Level V	The person is seldom able to communicate effectively, even with familiar people.

a The classification considers all forms of communication, including speech, gestures, facial expressions, and augmentative and alternative communication (AAC).

The CFCS was selected because it offers a brief, reliable, and ecologically valid measure applicable across communication modalities, allowing standardized comparison of functional communication abilities within the study cohort. It does not capture detailed speech, language, or interactional skills—necessitating complementary assessments for a comprehensive evaluation. Therefore, the Viking Speech Scale, VSS (39), was also used as well as the use of AAC. The VSS classify speech production from Level I (*speech is not affected by motor disorder*) to Level IV (*no understandable speech*). The use of aided or unaided AAC was noted. Unaided AAC comprises manual signs, gestures, and facial expressions (40). These modes of communication often require adequate motor control and communication partners who can interpret the communication. Aided AAC includes objects, pictures or written words used individually or as part of a low-tech communication system or high-tech speech-generating devices/apps, and can be accessed through pointing, head tracking, or eye gaze technology, for example. Information on previous school form was noted as a proxy for intellectual function as having followed a curriculum adapted for pupils with intellectual disability is possible only for individuals with an IQ below 70 in Sweden.

The RTP was developed based on theories of developmental psychology to summarize the transition process of young persons with CP and was used to indicate level of independence across everyday life areas (21). It consists of the domains: Education and employment, Finances, Housing, Leisure, Relationships, Sexuality (added to the original version to explore sexuality in the context of disability (41), Transportation, Care demands, Services and aids, and Rehabilitation services. The level of independence is categorized into one of three or four phases (varies across domains) of transition: “childhood”, “transition” or “adulthood”. The RTP has been reported to give a valid and reliable description of phases of transition for young persons with CP (19, 21, 42) and is available in Swedish (43). Although primarily designed for adolescents and young adults without intellectual disability, the RTP has been administrated in samples with more varied clinical characteristics (42) and ages (19). The RTP includes options such as “vocational training” and “parents or carers arrange transportation”, making it valid also to persons with developmental disabilities. Furthermore, phase 3 of the RTP domains is referred to as “adulthood” and include options relevant to many adults above 25 years of age, reflecting established adult roles.

### 2.4 Data collection

Adults who consented to participate, and those whose legal guardian gave consent on their behalf, were invited to a research visit comprising a clinical examination, an interview, and the completion of several questionnaires, including the RTP. Data were collected by a multi-professional team with extensive clinical experience of patients with CP. The levels of the GMFCS, MACS, CFCS, and VSS were determined during the interview, based on clinical observations and self- and/ or proxy reports. Self-response was obtained whenever possible and individual adaptations were made to facilitate the participation of adults with CCN and/ or intellectual disability. Adaptations included the location and scheduling of the research visit, as well as providing extra time for personal assistance with mobility, communication, and other daily needs during the data collection. The use of personal AAC was encouraged and there was an option to complete the questionnaires in an interview format using Talking Mats (44) with pictures supporting both comprehension and modes of answering. A proxy-response from someone who knew the adult well (parents, carers or personal assistants) was recorded for adults unable to complete the questionnaires despite adaptations. The proxies were instructed to answer on behalf of the adult with CP.

### 2.5 Data analysis

A dropout analysis using the Chi-square test for independence was conducted to control for differences in characteristics between those who responded and those who did not respond to the study invitation.

CFCS was dichotomized to “effective communication” (Levels I – II) and “non-effective communication” (Levels III – V), and VSS was dichotomized to “unaffected speech” (Levels I – II) and “reduced speech intelligibility” (Levels III – IV) due to small group sizes. The cut-off was made where both classifications distinguish between the communication effectiveness with unfamiliar communication partners and has been used in previous studies on young adults with CP (20). The categories of the RTP were handled as nominal variables. Regarding contact with rehabilitation services, the second response alternative, “no rehabilitation visits the last year”, could be selected both for persons in no need of rehabilitations services, as well as for persons with unmet needs. Therefore, this domain is only reported as a descriptive variable.

Sample characteristics were summarized as mean (*M*) and standard deviation (*SD*), or *n* and percentages. Non-parametric tests were used to explore group differences and associations between participant characteristics as there was an imbalance between the response options and several cells with small numbers of responses. Fisher-Freeman-Halton exact test was used to compare categorical variables. The Mann–Whitney *U* test and the Kruskal Wallis *H* test, followed by a *post hoc* pairwise comparisons with a Bonferroni adjusted alpha level, was used to compare continuous variables. Effect sizes (*ES*), degrees of freedom (*df*) and *p*-values are reported. Effect sizes were computed using Cramer's *V* on categorical variables, and Cohen's *d* on continuous variables. A two-tailed *p*-value of 0.05 or less defined statistical significance. Analyses were performed using IBM SPSS Statistics (Version 28). The total number of participants for each item and analysis is reported. No imputation was made where items were unanswered.

## 3 Results

### 3.1 Sample characteristics

A total of 571 study invitations were sent out and 145 adults (25%) agreed to participate. The drop-out analysis revealed that among the respondents, compared to non-respondents, there was a lower proportion of adults walking without aids (*x^2^* = 6.63, *df* = 1, *p* = .013) and a larger proportion of adults using wheelchair (*x^2^*= 6.10, *df* = 1, *p* = .014). There was no statistically significant difference between respondents and non-respondents related to intellectual disability (*x^2^*= 3.59, *df* = 1, *p* = .062).

The RTP was completed by 139 persons with CP (Table 2), mean age 30y 1mo (*SD* 5.85) of which 100 participants described themselves as having “effective communication” (CFCS Levels I-II) and 39 participants “non-effective communication” (CFCS Levels III-V). Proxy responses (*n* = 36, 26%) were equally common in men and women, and associated with lower levels of functional ability: GMFCS, *x^2^* (4, *n* = 139) = 56.30, *p* < .001, *ES* 0.64; MACS, *×^2^* (4, *n* = 139) = 60.75, *p* < .001, *ES* 0.66; CFCS, *x^2^* (4, *n* = 139) = 87.29, *p* < .001, *ES* 0.79; VSS, *x^2^* (3, *n* = 139) = 80.76, *p* < .001, *ES* 0.76). Of the 36 participants with proxy responses, 16 used AAC (44%). All proxy respondents had followed a curriculum adapted for pupils with intellectual disability.

**Table 2:** Demographic characteristics of participants (*n* = 139).

Participant Characteristics	Effective Communication^a^ *n* = 100	Non-effective communication^a^ *n* = 39	*P* Value^b^	Effect size^c^
Gender *n* (%)				
Male	51 (51)	23 (59)	.551	0.11
Female	46 (46)	16 (41)		
Nonbinary	3 (3)	0 (0)		
Age, M (SD)	31.49 (5.70)	26.97 (5.35)	<.001	0.70
School form *n* (%)				
Mainstream, non-adapted curriculum	69 (69)	1 (3)	<.001	0.60
Curriculum adapted for pupils with intellectual disability	31 (31)	38 (97)		
Rehabilitation Services *n* (%)				
Child rehabilitation	1 (1)	3 (8)	<.001	0.31
No rehabilitation visits in the past year	30 (30)	2 (5)		
Rehabilitation, adult department	69 (69)	34 (87)		
CP^d^ classification^e^ *n* (%)				
Unilateral spastic	40 (40)	1 (3)	<.001	0.48
Bilateral spastic	46 (46)	18 (46)		
Dyskinetic	11 (11)	19 (48)		
Ataxic	3 (3)	1 (3)		
GMFCS^f^ level *n* (%)				
I	47 (47)	1 (3)	<.001	0.77
II	25 (25)	2 (5)		
III	8 (8)	1 (3)		
IV	16 (16)	6 (15)		
V	4 (4)	29 (74)		
MACS^g^ level *n* (%)				
I	50 (50)	0 (0)	<.001	0.77
II	35 (35)	3 (8)		
III	6 (6)	4 (10)		
IV	4 (4)	8 (20)		
V	5 (5)	24 (62)		
Viking Speech Scale *n* (%)				
I	77 (77)	1 (3)	<.001	0.88
II	18 (18)	2 (5)		
III	3 (3)	5 (13)		
IV	2 (2)	31 (79)		
Respondent *n* (%)				
Self-response	94 (94)	9 (23)	<.001	0.73
Proxy-response	6 (6)	30 (77)		
AAC^h^ use *n* (%)				
No report of AAC	95 (95)	20 (51)	<.001	0.53
Unaided AAC	0 (0)	2 (5)		
Aided AAC	4 (4)	9 (23)		
Unaided and aided AAC	1 (1)	8 (21)		

a “Effective communication” refer to participants classified with Communication Function Classification System (CFCS) Levels I-II, and “Non-effective communication” to participants classified with CFCS Levels III to V.

b Comparison between effective and non-effective communicators were made using Fisher-Freeman-Halton exact test on categorical variables, and Mann–Whitney *U* test on continuous variables.

c Effect sizes were computed using Cramer's *V* on categorical variables, and Cohen's *d* on continuous variables.

d Cerebral Palsy.

e Classification according to Surveillance of Cerebral Palsy in Europe, SCPE.

f Gross Motor Function Classification System.

g Manual Ability Classification System.

h Augmentative and alternative communication.

**Figure 1:**
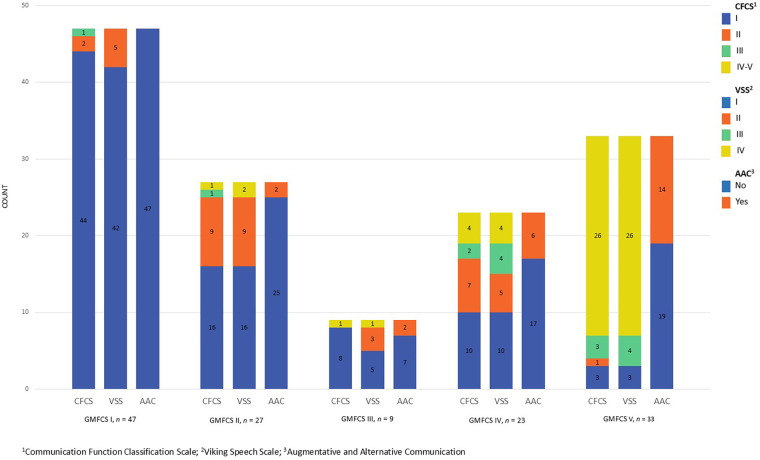
Communication abilities grouped by gross motor classification scale (*n* = 139).

Participants functioning at CFCS Levels I-II (“effective communication”) demonstrated significantly higher levels of gross motor function, manual ability, and speech production with moderate (>0.2 ≤ 0.6) to strong (>0.6) effect sizes for all significant differences. However, some participants functioning at GMFCS Levels I and II were noted to have higher levels of speech impairment (CFCS Levels III-V, “non-effective” communication), reduced speech intelligibility, and used AAC (Figure 1). All participants but one with non-effective communication had followed a school curriculum adapted for pupils with intellectual disabilities.

In the current sample, younger participants had lower levels of gross motor function [H (4, *n* = 139) = 14.97, *p* = .005]. *Post-hoc* analyses with a Bonferroni adjusted alpha level revealed differences between GMFCS Level I (*M* 31.38, *SD* 5.78) and Level V (*M* 27.45, *SD* 5.39; *p* = .049), and between Level II (*M* 32.78, *SD* 5.14) and Level V (*M* 27.45, *SD* 5.39; *p* = .005).

### 3.2 Description of independence according to Rotterdam transition profile

#### 3.2.1 Communication effectiveness

The highest level of independence for participants with effective and non-effective communication was reported in the RTP domain of Finances (93% and 90% respectively), Table 3. Effective communication was associated with higher levels of independence across all domains of the RTP. Strong effect sizes were noted in the domains Leisure, Transportation, Care demands, and Service and aids.

**Table 3:** Comparison between rotterdam transition profile and communication effectiveness, speech production, augmentative and alternative communication (AAC; *n* = 139).

Rotterdam Transition Profile	Communication Effectiveness^a^				Speech Production^b^				Use of AAC^c^			
	Effective comm-unication *n* (%)	Non -effective comm-unication *n* (%)	*P*-Value^d^	Effect size^e^	Un-affected speech *n* (%)	Reduced speech intelli-gibility *n* (%)	*P*-Value^d^	Effect size^e^	No AAC *n* (%)	AAC *n* (%)	*P*-Value^d^	Effect size^e^
Employment and education (*n* = 139)												
(0) Not in education or job	6 (6)	6 (15)	.007	0.30	5 (5)	7 (17)	.003	0.32	6 (5)	6 (25)	<.001	0.43
(1) General education	1 (1)	4 (10)			1 (1)	4 (10)			1 (1)	4 (17)		
(2) Vocational training, work placement	5 (5)	0 (0)			5 (5)	0 (0)			5 (4)	0 (0)		
(3) Paid job, volunteer work, sheltered employment	88 (88)	29 (75)			87 (89)	30 (73)			103 (90)	14 (58)		
Finances (*n* = 139)												
(1) Pocket money, clothing allowance	2 (2)	4 (10)	.043	0.22	2 (2)	4 (10)	.051	0.21	2 (2)	4 (17)	.013	0.29
(2) Job on the side, student grants	5 (5)	0 (0)			5 (5)	0 (0)			5 (4)	0 (0)		
(3) Economically independent: benefits or job income	93 (93)	35 (90)			91 (93)	37 (90)			108 (94)	20 (83)		
Housing (*n* = 139)												
(1) Living with parents, no responsibility for household activities	6 (6)	12 (31)	<.001	0.35	7 (7)	11 (27)	.008	0.27	10 (9)	8 (33)	.008	0.28
(2) Seeking housing, domestic training	7 (7)	0 (0)			6 (6)	1 (2)			6 (5)	1 (4)		
(3) Living independently	87 (87)	27 (69)			85 (87)	29 (71)			99 (86)	15 (63)		
Leisure (*n* = 137)												
(0) Parents or staff organises activities	9 (9)	28 (72)	<.001	0.64	12 (13)	25 (63)	<.001	0.52	27 (24)	10 (43)	.193	0.18
(1) The young adult organises leisure activities with peers at home	2 (2)	0 (0)			2 (2)	0 (0)			2 (2)	0 (0)		
(2) The young adult organises leisure activities with peers outside during daytime hours	9 (9)	2 (5)			9 (9)	2 (5)			9 (8)	2 (9)		
(3) The young adult goes out in the evening with peers	78 (80)	9 (23)			74 (76)	13 (32)			76 (66)	11 (48)		
Relationships (*n* = 133)												
(0) The young adult has no experience of dating	16 (17)	25 (68)	<.001	0.51	18 (19)	23 (59)	<.001	0.43	32 (29)	9 (41)	.314	0.16
(1) The young adult has experience of dating, but no courtship	13 (14)	2 (5)			12 (13)	3 (8)			13 (12)	2 (9)		
(2) The young adult has experience of courtship	20 (21)	6 (16)			18 (19)	8 (20)			20 (18)	6 (27)		
(3) The young adult has experience of a romantic relationship/ a partner	47 (49)	4 (11)			46 (49)	5 (13)			46 (41)	5 (23)		
Sexuality (*n* = 128)												
(0) The young adult has no experience of kissing	19 (20)	27 (82)	<.001	0.60	21 (22)	25 (73)	<.001	0.55	37 (33)	9 (53)	.018	0.28
(1) The young adult has experience of kissing	12 (12)	5 (15)			10 (11)	7 (21)			12 (11)	5 (29)		
(2) The young adult has experience of caressing underneath the clothes or cuddling nude	11 (12)	0 (0)			11 (12)	0 (0)			11 (10)	0 (0)		
(3) The young adult has experience of intercourse	53 (56)	1 (3)			52 (55)	2 (6)			51 (46)	3 (18)		
Transportation (*n* = 139)												
(1) Parents or staff transport the young adult	12 (12)	28 (72)	<.001	0.63	13 (13)	27 (66)	<.001	0.55	27 (23)	13 (54)	.004	0.28
(2) Parents or staff arrange transportation	7 (7)	5 (13)			8 (8)	4 (10)			9 (8)	3 (13)		
(3) The young adult arrange transportation independently	81 (81)	6 (15)			77 (79)	10 (24)			79 (69)	8 (33)		
Care demands (*n* = 138)												
(1) Parents or staff formulate care demands	5 (5)	27 (69)	<.001	0.72	5 (5)	27 (66)	<.001	0.69	20 (17)	12 (50)	<.001	0.37
(2) Parents or staff and young adult formulate care demands together	27 (27)	10 (26)			27 (28)	10 (24)			28 (25)	9 (38)		
(3) Young adult formulates care demands	67 (68)	2 (5)			65 (67)	4 (10)			66 (58)	3 (12)		
Service and aids (*n* = 138)												
(1) Parents or staff apply for service and aids	14 (14)	32 (82)	<.001	0.67	16 (16)	30 (73)	<.001	0.58	31 (27)	15 (63)	<.001	0.35
(2) The young adult learns the procedures	23 (23)	6 (15)			21 (22)	8 (20)			22 (19)	7 (29)		
(3) The young adult applies for service and aids independently	62 (63)	1 (2)			60 (62)	3 (7)			61 (54)	2 (8)		

a “Effective communication” refer to participants classified with Communication Function Classification System (CFCS) Levels I-II, and “Non-effective communication” to participants classified with CFCS Levels III to V.

b “Unaffected speech” refers to participants classified with Viking Speech Scale (VSS) Levels I - II, and “Reduced speech intelligibility” refers to participants classified with VSS Levels III - IV.

c Aided and non-aided augmentative and alternative communication, AAC, was grouped under “AAC”.

d P-values refer to Fisher-Freeman-Halton exact test.

e Effect sizes were computed using Cramer's *V.*

Participants functioning at GMFCS Levels I – II and/or MACS Levels I – II reported higher levels of independence in all RTP domains compared to participants functioning at GMFCS Levels III – V and/or MACS Levels III – V, showing moderate effect sizes (Supplementary Table S1). The associations between motor function and the domains “Employment and education” and “Finances” were slightly stronger compared to the association between communication effectiveness and the same domains (*ES* 0.36 and 0.29 compared to *ES* 0.30 and 0.22). The associations were weaker between motor function and the seven other RTP domains, compared to the associations with communication effectiveness. In Supplementary Table S2, levels of independence were compared between participants who had followed a mainstream, non-adapted school curriculum and participants who had followed a curriculum adapted for pupils with intellectual disability. Having followed a non-adapted school curriculum was associated with higher levels of independence across all RTP domains. The reported independence levels of the RTP were similar when comparing school curriculum and communication effectiveness except for the domain “Service and aids” where non-effective communication seemed to have a greater negative impact on independence (*ES* 0.67 compared to 0.31). Comparing self-respondents with proxy-respondents, self-respondents reported higher levels of independence across all RTP domains (Supplementary Table S2). Strong effect sizes were noted in four domains: Leisure, Transportation, Care demands, and Service and aids.

Age in relation to the RTP is reported in Table 4. Participants living with their parents were younger than participants living independently in both the effective and the non-effective communication group. For participants with effective communication a higher level of independence was associated with older age within the Finance domain. For participants with non-effective communication, higher levels of independence were associated with older age within the Employment and Education domain.

**Table 4:** Associations between Age and rotterdam transition profile grouped by communication effectiveness (*n* = 139).

Rotterdam transition profile	Effective communication^a^ (*n* = 100)					Non-effective communication^a^ (*n* = 39)				
	N	Mean (*SD*)	Chi-Square (*df)*	*P*-Value^b^	Effect size^c^	N	Mean (*SD*)	Chi-Square (*df*)	PValue^b^	Effect size^c^
Employment and education, (*n* = 139)										
(0) Not in education or job	6 (6)	31.33 (6.80)	5.75 (3)	.125	0.03	6 (15)	26.67 (4.46)	−18.61 (2)	.006^d^	0.57
(1) General education	1 (1)	23 (-)				4 (10)	19.75 (1.5)			
(2) Vocational training, work placement	5 (5)	26.60 (6.66)				0 (0)	-			
(3) Paid job, volunteer work, sheltered employment	88 (88)	31.87 (5.47)				29 (75)	28.03 (5.14)			
Finances (*n* = 139)										
(1) Pocket money, clothing allowance	2 (2)	26.50 (4.95)	−42.30 (2)	.004^e^	0.42	4 (10)	27.25 (10.15)	.005 (1)	.944	0.03
(2) Job on the side, student grants	5 (5)	23.20 (2.60)				0 (0)	-			
(3) Economically independent: benefits or job income	93 (93)	32.04 (5.46)				35 (90)	26.94 (4.79)			
Housing (*n* = 139)										
(1) Living with parents, no responsibility for household activities	6 (6)	23.17 (2.71)	- 45.62 (2) - 45.20 (2)	<.001^f^ <.001^g^	0.45	12 (31)	22.08 (3.50)	16.48 (1)	<.001^f^	0.48
(2) Seeking housing, domestic training	7 (7)	23.71 (4.61)				0 (0)	-			
(3) Living independently	87 (87)	32.69 (4.93)				27 (69)	29.15 (4.56)			
Leisure (*n* = 137)										
(0) Parents or staff organises activities	9 (9)	31.11 (5.65)	3.80 (3)	.285	0.02	28 (72)	26.68 (5.19)	4.73 (2)	.094	0.08
(1) The young adult organises leisure activities with peers at home	2 (2)	32.50 (13.44)				0 (0)	-			
(2) The young adult organises leisure activities with peers outside during daytime hours	9 (9)	28.33 (4.3)				2 (5)	21.00 (0)			
(3) The young adult goes out in the evening with peers	78 (78)	31.96 (5.70)				9 (23)	29.22 (5.52)			
Relationships (*n* = 133)										
(0) The young adult has no experience of dating	16 (17)	29.94 (6.64)	8.20 (3)	.067	0.06	25 (68)	26.64 (5.11)	3.79 (3)	.285	0.02
(1) The young adult has experience of dating, but no courtship	13 (13)	32.15 (5.74)				2 (5)	28.50 (4.95)			
(2) The young adult has experience of courtship	20 (21)	28.80 (5.94)				6 (16)	24.83 (5.42)			
(3) The young adult has experience of a romantic relationship/ a partner	47 (49)	32.96 (4.81)				4 (11)	31.75 (7.27)			
Sexuality (*n* = 128)										
(0) The young adult has no experience of kissing	19 (20)	31.37 (5.75)	1.01 (3)	.799	0.02	27 (82)	27.07 (5.16)	1.94 (2)	.379	0.002
(1) The young adult has experience of kissing	12 (13)	31.83 (5.65)				5 (15)	26.60 (6.35)			
(2) The young adult has experience of caressing underneath the clothes or cuddling nude	11 (11)	29.64 (7.30)				0 (0)	-			
(3) The young adult has experience of intercourse	53 (56)	32.11 (5.37)				1 (3)	34 (-)			
Transportation (*n* = 139)										
(1) Parents or staff transport the young adult	12 (12)	30.92 (7.80)	1.11 (2)	.574	0.001	28 (72)	26.46 (4.96)	4.64 (2)	.098	0.07
(2) Parents or staff arrange transportation	7 (7)	29.86 (4.26)				5 (13)	24.80 (5.93)			
(3) The young adult arrange transportation independently	81 (81)	31.72 (5.49)				6 (15)	31.17 (5.42)			
Care demands (*n* = 138)										
(1) Parents or staff formulate care demands	5 (5)	29.00 (6.44)	1.88 (2)	.391	0.001	27 (69)	26.33 (5.19)	1.46 (2)	.481	0.02
(2) Parents or staff and young adult formulate care demands together	27 (27)	30.65 (5.91)				10 (26)	28.20 (5.87)			
(3) Young adult formulates care demands	67 (68)	31.96 (5.55)				2 (5)	29.50 (6.36)			
Service and aids (*n* = 138)										
(1) Parents or staff apply for service and aids	14 (14)	29.64 (4.80)	2.29 (2)	.318	0.003	32 (82)	26.87 (5.28)	1.86 (2)	.396	0.004
(2) The young adult learns the procedures	23 (23)	31.52 (5.26)				6 (15)	26.33 (5.85)			
(3) The young adult applies for service and aids independently	62 (63)	32.03 (5.97)				1 (3)	34 (-)			

a “Effective communication” refer to participants classified with Communication Function Classification System (CFCS) Levels I-II, and “Non-effective communication” to participants classified with CFCS Levels III to V.

b *P*-values refer to the Kruskal Wallis H test followed by a *post hoc* pairwise comparisons with a Bonferroni adjusted alpha level.

c Effect sizes were calculated using Eta squared (*n^2^*).

d Significant difference in age between “General education” and “Paid job, volunteer work, sheltered employment”.

e Significant difference in age between “Job on the side, student grants” and “Economically independent”.

f Significant difference in age between “Living with parents” and “Living independently”.

g Significant difference in age between “Living with parents” and “Seeking housing, domestic training”.

#### 3.2.2 Speech production

Speech production was explored as a more specific communication variable than the broad CFCS classifications. Participants described as having an unimpaired speech reported higher levels of independence across domains of the RTP, except for the Finances domain, compared to participants with reduced speech intelligibility, with predominantly moderate effect sizes (Table 3). A strong effect size was noted for the association between speech production and Care demands.

#### 3.2.3 Use of AAC

A total of 5 participants with effective communication and 19 with non-effective communication used AAC. These 24 participants corresponded to 17% of the total sample. Participants using AAC (of which 67% had proxy-respondents) reported lower levels of independence compared to participants not using AAC. However, in the domains of Leisure and Relationships, the use of AAC supported independence (Table 3).

## 4 Discussion

This study describes independence in everyday life areas for persons with cerebral palsy from a communication point-of-view. We explored independence related to communication effectiveness, speech production, and the use of AAC. For these three aspects findings were in accordance with our hypotheses.

Overall, the levels of independence among the participants were highly variable: high levels of independence in one life area coincided with a lack of independence in other areas. In the domain Finances, over 90 per cent of all participants reported the highest level of independence. In contrast, less than 65 per cent reported the highest level of independence the domains of Relationships, Sexuality, and Service and Aids. Such variability has been described previously (19, 21, 42). Similar to the Dutch studies (19, 21), financial independence was reported also among participants with higher functional impairment, likely due to services and financial subsidies within the Swedish welfare system. Low levels of independence were reported in the Relationships and Sexuality domains: nearly one out of five participants with effective communication had no experience of dating (the lowest level in the Relationships domain) or kissing (the lowest level the Sexuality domain). Within the group of participants with non-effective communication, corresponding proportions were 68 per cent and 82 per cent respectively. A total of 56 per cent of the participants with effective communication and 1 participant (3%) with non-effective communication had experience of sex. These numbers can be contrasted with the general population in Sweden where 81 per cent of people between 16 and 29 have had sex at least once (45). The results of the present study display even less sexual independence than the study by Wiegerink et al. (46), which only included adults with CP without intellectual disability. In addition to low reported levels of independence, the two domains of Relationships and Sexuality also had the most missing responses, sometimes commented on as “not relevant” (often by proxies). Previous research has shown that the sexual development of young persons with physical disabilities is often neglected, especially for those relying on assistance from others in their activities of daily living (41, 46). Consequently, young persons with physical disabilities may have unmet needs with respect to the development of romantic and sexual relationships. The preset study adds to this body of research, complementing it with a communicative perspective. The low levels of independence and the assumption of intimacy as irrelevant reported in the current study, especially among participants with non-effective communication, need to be considered both in the light of opportunities for persons with CP to explore one's sexuality and develop intimate relationships, and the voluntariness in sexual activity and the ability to give consent. The noted lack of AAC use among study participants with non-effective communication could be an obstacle for both aspects. Professionals working in rehabilitation and other services should be aware of this variability in independence related to intimate relationships and provide adequate, personalized support.

The study's communication perspective highlights several important aspects. Firstly, nearly one third of the adults were described as having non-effective communication and they were represented across all levels of gross motor function and across all CP types. AAC users had gross motor function ranging from GMFCS Level II to V. Thus, CCN are common in adults with CP and regardless of motor function, communication needs must be met to support independence in everyday life areas. Also, future studies need to routinely include participants with CCN to more fully understand adult life when living with CP.

Secondly, lower levels of independence across most domains of everyday life were associated with having non-effective communication and impaired speech intelligibility. The many stronger effect sizes noted in the associations between communication effectiveness and independence compared to associations with other personal characteristics such as gross motor function, school curriculum, and age further suggest that communication function is a variable imperative to include when trying to deepen the understanding of independence in adults with CP. The association between less independence in most of the explored domains and having non-effective communication and impaired speech intelligibility is in line with other studies (47, 48). The results of this study expand on these previous findings, emphasizing that CCN, along with greater motor impairment, are considerable barriers to achieving higher education and accessing employment for adults with CP (6, 47). Participants with non-effective communication and overall greater functional impairment were also more often represented by proxy-responses, thus dependent on and vulnerable to others' interpretation of their needs, experiences and wishes. In the present study, strongest effect sizes were noted in the domains Care demands and Service and aids, a concerning finding as persons with CP have a significantly increased risk of multiple health conditions, more frequent hospital visits, and longer hospital stays compared to persons without CP (49). These findings underscore the need for both proxies and healthcare professionals to be aware of the responsibility that comes with being the interpreter of another person's needs and wishes.

The healthcare dialogue, including essential exchanges between a patient and a healthcare professional, is dependent on communication competence related to understanding and expressing preferences and needs of care, health-related goals and evaluation of treatment, as well as activity and participation choices (50). Common themes in previous research on transition from child- to adult healthcare settings are lack of preparedness, difficulty navigating the adult system, and lack of knowledgeable service providers (16–18). In the current study, 69 per cent of the participants with non-effective communication had their care requirements formulated by parents of staff, and parents or staff applied for service and aids for 82 per cent of these participants. Improved communication competence, both in persons with CP and their communication partners, could increase autonomy in relation to one's own health without discrimination based on disability.

Thirdly, the 24 participants using AAC were as independent in the domains of Leisure and Relationships as the 115 participants not using AAC, underscoring the importance of meaningful and effective AAC to support social relationships. Parallel to previous findings, AAC interventions can alleviate the impact of communication impairments on social outcomes in adults with CP through providing opportunities for interaction with others, access to the community on one's own, and reduce feelings of loneliness (6, 51). However, this finding stresses the significance of AAC systems that are functioning, known by communication partners, and in use (6). A total of 39 participants in the current study were described as having non-effective communication, yet only 19 (49%) of these reported using AAC, a finding signaling unmet needs related to communication support. Increased attention to the provision of AAC systems with adult-specific vocabulary along with individual support to develop literacy skills and increased knowledge of AAC among people in the environment are important steps for making it possible for adults with CP and CCN to pursue their life goals.

Lastly, a comment needs to be made concerning the RTP instrument. The distinction between “independence” and “autonomy” when describing transition phases was found to be inconsistent across and within the items of the instrument. For example, the highest level of independence within the employment domain includes having a paid job, doing volunteer work or sheltered employment which decreases the clarity of the instrument and warrant further conceptual development. Although these two concepts, independence and autonomy, are related, they refer to different concepts with important implications for research and practice (52). Autonomy refers to an individual's capacity for self-directed decision-making based on personal values and preferences, regardless of whether assistance is required to implement those choices. Independence, in contrast, denotes the ability to perform tasks without external support, emphasizing functional capability rather than decision-making. Differentiating these concepts both in practice and in research is crucial because a person may retain autonomy even when lacking independence, as seen in contexts such as disability support or healthcare. Recognizing this distinction ensures that interventions and policies prioritize respect for personal agency and rights, rather than equating autonomy solely with physical self-sufficiency (5, 53).

### 4.1 Limitations

A number of participants had their data reported by proxies, despite adaptations of the data collection procedures being available, and this may limit the interpretation of the findings. However, proxy responses have been widely used in order to include children and people with communication disorders, for example regarding quality of life (4, 54).

Other limitations of the study are the skewed sample related to gross motor function and age, the extended data collection period, and the cross-sectional design of the study. Respondents displayed higher levels of gross motor impairment compared to non-respondents, and participants with effective communication (and lower levels of impairment overall) were older than participants with non-effective communication which means the findings coupled to age and independence needs to be interpreted with caution. Many younger adults with CP and higher levels of gross motor function, manual ability, and speech production no longer have contact with habilitation services and therefore may be less motivated to participate. A similar pattern of recruitment bias has been noted previously (4). Younger adults still in contact with habilitation services were given information of the study firsthand by healthcare professionals, thus presumed to have a lower threshold to participate. Another consideration relating to the sample is the data collection spanning eight years, from 2015 to 2023. This strategy allowed for recruitment of participants with a variety in CP types, functional abilities and a sufficient sample size. The broad time span may have captured descriptions and differences that are no longer of relevance or may have missed out on more contemporary issues. However, there have been no major changes within the Swedish welfare system during the study period related to the areas explored in the RTP. The study took place during the COVID19 pandemic, which further extended the data collection due to restrictions on movement, and measures called for by the Public Health Agency of Sweden, to reduce potential risk for participants and researchers. Only 8 study visits took place between March 2020 and March 2022, thus had negligible impact on the study findings. The mean age of participants included before the pandemic outbreak was 32 years compared to 29 years after.

The descriptive, cross-sectional study design gives a snapshot of level of independence, but no information on change over time or potential causal effects. Transition to adulthood includes parallel processes related to independence extending over several years and everyday contexts. Also, although the injuries to the developing brain causing CP are non-progressive, the impact and presentation of impairments do change over time. A longitudinal study design would depict a more dynamic view of transition processes and independence, and explore to what extent such processes are delayed, elongated or maybe not present at all.

### 4.2 Practical implications

One key finding is the additional challenges in achieving independence for adults with CP and CCN. Practitioners need to be aware of these challenges and continuously assess, provide and follow up on tailored AAC systems for adults with CCN, and promote increased knowledge in communication supporting strategies among staff working with these adults. As suggested by Dark et al (55) speech-language pathologists can play a crucial role in supporting adults with CP to maintain control and self-determination in their lives in the event of communication changes over the life course. Additionally, including participants in a wider age span than the typical transition-to-adulthood- period reveals important findings. Challenges with independence in everyday life areas usually linked to adapting to adult roles persist beyond the mid-twenties, regardless of communication effectiveness. Thus, support should be provided in areas associated with transition to adulthood over an extended period, to facilitate adults to engage in things they have to and want to do, for example participation in leisure activities, formulation of healthcare needs, and finding a meaningful occupation. The current findings also underscore that social workers and educators need to initiate and support conversations about romantic relationships and sexuality with adolescents and adults as well as with any involved carers.

### 4.3 Concluding remarks

Participants with effective communication and unimpaired speech were more independent in everyday life areas than participants with non-effective communication, and impaired speech intelligibility. The use of ACC showed encouraging decrease in the difference of independence in the domains of Leisure and Relationships between participants with non-effective communication compared to those with effective communication. Low levels of independence related to healthcare services and intimate relationships were reported, especially for participants with non-effective communication.

Persons with inconsistent and/ or non-verbal communication have often been excluded in studies on independence and transition to adulthood, thus limiting the transferability of prior research to persons with CP and complex communication needs. By promoting the use of AAC, offering modified answering forms, and being attentive in how data collection could be adapted, this study provides a picture of independence among adults with CP with a more varied functional ability. These strategies are also valuable in clinical work when supporting young adults finding their roles in various formal and informal everyday life contexts.

## Data Availability

The datasets presented in this article are not readily available because the data is archived in Secured Data Repository at Sahlgrenska University Hospital, Gothenburg, Sweden. To protect the privacy of the participants, data cannot be shared publicly . Requests to access the datasets should be directed to the corresponding author.
