# Supplementary material for: The impact of communication abilities on independence in everyday life—a cross-sectional study of adults with cerebral palsy
Source: Front Rehabil Sci. 2026 May 13;7:1710509. doi: 10.3389/fresc.2026.1710509 (PMC13212311; doi:10.3389/fresc.2026.1710509)
Supplement: Supplementary file 1 [file Datasheet1.docx]

**Supplementary material**

**Tables**

**Table S1.** Comparison Between Gross Motor Function, Manual Functioning, and Rotterdam Transition Profile (*n* = 139).

**Table** **S2.** Comparison Between School Curriculum, Respondent and Rotterdam Transition Profile (*n* = 139).

**Table S1.** Comparison Between Gross Motor Function, Manual Functioning, and Rotterdam Transition Profile (*n* = 139).

| Rotterdam Transition Profile | Gross Motor Function^1^ | | | | Manual Function^2^ | | | |
| --- | --- | --- | --- | --- | --- | --- | --- | --- |
|  | I - II  *n* (%) | III - IV  *n* (%) | P Value^3^ | Effect Size^2^ | I - II  *n* (%) | III - IV  *n* (%) | P Value | Effect Size |
| Employment and Education, (*n* = 139) | | | | | | | | |
| (0) Not in education or job | 1 (1) | 11 (17) | <.001 | .36 | 3 (3) | 9 (18) | .003 | .31 |
| (1) General education | 0 (0) | 5 (8) |  |  | 1 (1) | 4 (8) |  |  |
| (2) Vocational training, work placement | 3 (4) | 2 (3) |  |  | 4 (5) | 1 (2) |  |  |
| (3) Paid job, volunteer work, sheltered employment | 71 (95) | 46 (72) |  |  | 80 (91) | 37 (72) |  |  |
| Finances (*n* = 139) | | | | | | | | |
| (1) Pocket money, clothing allowance | 0 (0) | 6 (9) | <.001 | .29 | 1 (1) | 5 (10) | .012 | .25 |
| (2) Job on the side, student grants | 5 (7) | 0 (0) |  |  | 5 (6) | 0 (0) |  |  |
| (3) Economically independent: benefits or job income | 70 (93) | 58 (91) |  |  | 82 (93) | 46 (90) |  |  |
| Housing (*n* = 139) | | | | | | | | |
| (1) Living with parents or staff, no responsibility for household activities | 2 (3) | 16 (25) | <.001 | .35 | 4 (4) | 14 (27) | <.001 | .34 |
| (2) Seeking housing, domestic training | 6 (8) | 1 (2) |  |  | 6 (7) | 1 (2) |  |  |
| (3) Living independently | 67 (89) | 47 (73) |  |  | 78 (87) | 36 (71) |  |  |
| Leisure (*n* = 137) | | | | | | | | |
| (0) Caregivers or staff organises activities | 6 (8) | 31 (50) | <.001 | .48 | 8 (9) | 29 (58) | <.001 | .53 |
| (1) The young adult organises leisure activities with peers at home | 1 (1) | 1 (2) |  |  | 2 (3) | 0 (0) |  |  |
| (2) The young adult organises leisure activities with peers outside during daytime hours | 9 (12) | 2 (3) |  |  | 9 (10) | 2 (4) |  |  |
| (3) The young adult goes out in the evening with peers | 59 (79) | 28 (45) |  |  | 68 (78) | 19 (38) |  |  |
| Relationships (*n* = 133) | | | | | | | | |
| (0) The young adult has no experience of dating | 10 (13) | 31 (53) | <.001 | .47 | 14 (16.5) | 27 (56) | <.001 | .49 |
| (1) The young adult has experience of dating, but no courtship | 12 (16) | 3 (5) |  |  | 12 (14) | 3 (6) |  |  |
| (2) The young adult has experience of courtship | 14 (19) | 12 (21) |  |  | 14 (16.5) | 12 (25) |  |  |
| (3) The young adult has experience of a romantic relationship/ a partner | 39 (52) | 12 (21) |  |  | 45 (53) | 6 (13) |  |  |
| Sexuality (*n* = 128) | | | | | | | | |
| (0) The young adult has no experience of kissing | 13 (18) | 33 (61) | <.001 | .49 | 17 (20) | 29 (68) | <.001 | .52 |
| (1) The young adult has experience of kissing | 10 (13) | 7 (13) |  |  | 10 (12) | 7 (16) |  |  |
| (2) The young adult has experience of caressing underneath the clothes or cuddling nude | 6 (8) | 5 (9) |  |  | 8 (9) | 3 (7) |  |  |
| (3) The young adult has experience of intercourse | 45 (61) | 9 (17) |  |  | 50 (59) | 4 (9) |  |  |
| Transportation (*n* = 139) | | | | | | | | |
| (1) Parents or staff transport the young adult | 7 (9) | 33 (52) | <.001 | .48 | 9 (10) | 31 (61) | <.001 | .55 |
| (2) Parents or staff arrange transportation | 6 (8) | 6 (9) |  |  | 8 (9) | 4 (8) |  |  |
| (3) The young adult arrange transportation independently | 62 (83) | 25 (39) |  |  | 71 (81) | 16 (31) |  |  |
| Care demands (*n* = 138) | | | | | | | | |
| (1) Parents or staff formulate care demands | 2 (3) | 30 (48) | <.001 | .59 | 3 (3) | 29 (57) | <.001 | .64 |
| (2) Parents or staff and young adult formulate care demands together | 18 (24) | 19 (30) |  |  | 24 (28) | 13 (25) |  |  |
| (3) Young adult formulates care demands | 55 (73) | 14 (22) |  |  | 60 (69) | 9 (18) |  |  |
| Service and aids (*n* = 138) | | | | | | | | |
| (1) Parents or staff apply for service and aids | 9 (12) | 37 (58) | <.001 | .56 | 12 (14) | 34 (67) | <.001 | .57 |
| (2) The young adult learns the procedures | 13 (18) | 16 (25) |  |  | 19 (22) | 10 (20) |  |  |
| (3) The young adult applies for service and aids independently | 52 (70) | 11 (17) |  |  | 56 (64) | 7 (13) |  |  |

^1^Gross motor function refers to classification according to Gross Motor Classification Scale, GMFCS.

^2^Manual function refers to classification according to Manual Ability Classification Scale, MACS.

^3^*P*-values refer to Fisher-Freeman-Halton exact test.

^4^Effect sizes were computed using Cramer’s *V*.

**Table S2.** Comparison Between School Curriculum, Respondent and Rotterdam Transition Profile, (*n* = 139).

| Rotterdam Transition Profile | School Curriculum | | | | Respondent | | | |
| --- | --- | --- | --- | --- | --- | --- | --- | --- |
|  | Mainstream non-adapted curriculum  *n* (%) | Adapted for pupils with intellectual disability  *n* (%) | P Value^1^ | Effect Size^2^ | Self-respondent  *n* (%) | Proxy-respondent  *n* (%) | P Value | Effect Size |
| Employment and Education, (*n* = 139) | | | | | | | | |
| (0) Not in education or job | 3 (4) | 9 (13) | .010 | 0.28 | 5 (5) | 7 (19) | <.001 | 0.35 |
| (1) General education | 0 | 7 |  |  | 1 (1) | 4 (11) |  |  |
| (2) Vocational training, work placement | 4 (6) | 1 (2) |  |  | 5 (5) | 0 (0) |  |  |
| (3) Paid job, volunteer work, sheltered employment | 63 (90) | 54 (78) |  |  | 92 (89) | 25 (70) |  |  |
| Finances (*n* = 139) | | | | | | | | |
| (1) Pocket money, clothing allowance | 0 (0) | 6 (9) | <.001 | 0.28 | 2 (2) | 4 (11) | .032 | 0.22 |
| (2) Job on the side, student grants | 5 (7) | 0 (0) |  |  | 5 (5) | 0 (0) |  |  |
| (3) Economically independent: benefits or job income | 65 (93) | 63 (91) |  |  | 96 (93) | 32 (89) |  |  |
| Housing (*n* = 139) | | | | | | | | |
| (1) Living with parents or staff, no responsibility for household activities | 3 (4) | 15 (22) | .006 | 0.26 | 7 (7) | 11 (31) | .002 | 0.31 |
| (2) Seeking housing, domestic training | 4 (6) | 3 (4) |  |  | 6 (6) | 1 (3) |  |  |
| (3) Living independently | 63 (90) | 51 (74) |  |  | 90 (87) | 24 (66) |  |  |
| Leisure (*n* = 137) | | | | | | | | |
| (0) Caregivers or staff organises activities | 1 (1.5) | 36 (52) | <.001 | 0.60 | 9 (9) | 28 (78) | <.001 | 0.70 |
| (1) The young adult organises leisure activities with peers at home | 1 (1.5) | 1 (2) |  |  | 2 (2) | 0 (0) |  |  |
| (2) The young adult organises leisure activities with peers outside during daytime hours | 4 (6) | 7 (10) |  |  | 8 (8) | 3 (8) |  |  |
| (3) The young adult goes out in the evening with peers | 62 (91) | 25 (36) |  |  | 82 (81) | 5 (14) |  |  |
| Relationships (*n* = 133) | | | | | | | | |
| (0) The young adult has no experience of dating | 9 (13) | 32 (50) | <.001 | 0.47 | 17 (17) | 24 (71) | <.001 | 0.52 |
| (1) The young adult has experience of dating, but no courtship | 5 (7) | 10 (16) |  |  | 13 (13) | 2 (6) |  |  |
| (2) The young adult has experience of courtship | 17 (25) | 9 (14) |  |  | 21 (21) | 5 (14) |  |  |
| (3) The young adult has experience of a romantic relationship/ a partner | 38 (55) | 13 (20) |  |  | 48 (49) | 3 (9) |  |  |
| Sexuality (*n* = 128) | | | | | | | | |
| (0) The young adult has no experience of kissing | 8 (12) | 38 (61) | <.001 | 0.64 | 20 (21) | 26 (81) | <.001 | 0.57 |
| (1) The young adult has experience of kissing | 5 (8) | 12 (20) |  |  | 13 (14) | 4 (13) |  |  |
| (2) The young adult has experience of caressing underneath the clothes or cuddling nude | 6 (9) | 5 (8) |  |  | 11 (11) | 0 (0) |  |  |
| (3) The young adult has experience of intercourse | 47 (71) | 7 (11) |  |  | 52 (54) | 2 (6) |  |  |
| Transportation (*n* = 139) | | | | | | | | |
| (1) Parents or staff transport the young adult | 4 (6) | 36 (52) | <.001 | 0.66 | 12 (12) | 28 (78) | <.001 | 0.71 |
| (2) Parents or staff arrange transportation | 0 (0) | 12 (17) |  |  | 6 (6) | 6 (17) |  |  |
| (3) The young adult arrange transportation independently | 66 (94) | 21 (31) |  |  | 85 (82) | 2 (5) |  |  |
| Care demands (*n* = 138) | | | | | | | | |
| (1) Parents or staff formulate care demands | 0 (0) | 32 (47) | <.001 | 0.75 | 5 (5) | 27 (75) | <.001 | 0.75 |
| (2) Parents or staff and young adult formulate care demands together | 10 (14) | 27 (40) |  |  | 29 (28) | 8 (22) |  |  |
| (3) Young adult formulates care demands | 60 (86) | 9 (13) |  |  | 68 (67) | 1 (3) |  |  |
| Service and aids (*n* = 138) | | | | | | | | |
| (1) Parents or staff apply for service and aids | 2 (3) | 44 (65) | <.001 | 0.31 | 14 (14) | 32 (89) | <.001 | 0.70 |
| (2) The young adult learns the procedures | 10 (14) | 19 (28) |  |  | 26 (25 | 3 (8) |  |  |
| (3) The young adult applies for service and aids independently | 58 (83) | 5 (7) |  |  | 62 (61) | 1 (3) |  |  |

^1^*P*-values refer to Fisher-Freeman-Halton exact test.

^2^Effect sizes were computed using Cramer’s *V*.
